# Resting State Network Segregation Modulates Age-Related Differences in Language Production

**DOI:** 10.1162/nol_a_00106

**Published:** 2023-06-13

**Authors:** Haoyun Zhang, Michele T. Diaz

**Affiliations:** Centre for Cognitive and Brain Sciences, University of Macau, Macau SAR, China; Department of Psychology, Pennsylvania State University, University Park, PA, USA

**Keywords:** cognitive aging, language production, network segregation, resting state functional connectivity

## Abstract

Older adults typically exhibit decline in language production. However, how the brain supports or fails to support these processes is unclear. Moreover, there are competing hypotheses about the nature of age-related neural changes and whether age-related increases in neural activity reflect compensation or a decline in neural efficiency. In the current study, we investigated the neural bases of language production focusing on resting state functional connectivity. We hypothesized that language production performance, functional connectivity, and their relationship would differ as a function of age. Consistent with prior work, older age was associated with worse language production performance. Functional connectivity analyses showed that network segregation within the left hemisphere language network was maintained across adulthood. However, increased age was associated with lower whole brain network segregation. Moreover, network segregation was related to language production ability. In both network analyses, there were significant interactions with age—higher network segregation was associated with better language production abilities for younger and middle-aged adults, but not for older adults. Interestingly, there was a stronger relationship between language production and the whole brain network segregation than between production and the language network. These results highlight the utility of network segregation measures as an index of brain function, with higher network segregation associated with better language production ability. Moreover, these results are consistent with stability in the left hemisphere language network across adulthood and suggest that dedifferentiation among brain networks, outside of the language network, is a hallmark of aging and may contribute to age-related language production difficulties.

## INTRODUCTION

Language is one of the most common and universal features of human society. However, as people age, they often face some decline in certain aspects of language, particularly in spoken language production (i.e., speech; Burke & Shafto, 2008; Diaz et al., 2016). For example, older adults speak more slowly in a variety of situations (Duchin & Mysak, 1987; Mortensen et al., 2006; Spieler & Griffin, 2006), have increased retrieval failures (e.g., Burke et al., 1991; Zhang et al., 2019), produce more filler words (Horton et al., 2010), produce more omissions (MacKay & James, 2004), produce more disfluent speech (Bortfeld et al., 2001; Obler & Albert, 1981), and produce less grammatically complex speech (e.g., Kemper et al., 2003). Several hypotheses have been proposed that these age differences in production may be due to declines in phonological processing (Burke et al., 1991; Burke & Shafto, 2008), aspects of executive function (Hasher et al., 1991; Hoffman et al., 2018; Lustig et al., 2007), or processing speed (Salthouse, 1996, 2000).

Although these behavioral age-related differences in language production have been commonly observed, how the brain supports or fails to support these processes is still not clear. Moreover, there are competing hypotheses about the nature of age-related neural changes. One prominent account is the dedifferentiation hypothesis, which suggests that age-related increases in neural activity reflect a decline in neural efficiency (Li et al., 2001). Others have suggested that age-related increases in neural activity are compensatory (Cabeza et al., 2018). One approach to investigating brain function is with resting state functional connectivity (RSFC), which examines how brain regions work together and thus how they form functional networks (Biswal et al., 1995; Harmelech & Malach, 2013). While RSFC can be used to investigate specific local networks, it can also measure many networks, thereby capturing a whole brain profile that can be used to investigate how overall brain function relates to cognition. Although studies have reported that more efficient brain networks are associated with enhanced cognition, few studies have focused on language production. Therefore, the current study investigated the relationship between RSFC and age-related differences in spoken language production, using network segregation, a measure that captures both within network and between network connectivities. We hypothesized that more segregated brain networks would be associated with enhanced language production and that this relationship would be modulated by age such that older adults would show less differentiated networks and weaker brain–behavior relationships.

Age-related behavioral differences in cognition and spoken language production are commonly observed, and this may be related to age-related differences in the brain. Even in the absence of disease, some degree of neural decline is a natural aspect of aging. For example, as people age they often show structural changes in the brain, such as decreased gray matter volume (Raz et al., 2005; Sowell et al., 2004) and decreased white matter integrity (Bartzokis et al., 2012; Imperati et al., 2011; Mwangi et al., 2013). In addition to structural brain changes, older and younger adults also exhibit different patterns of task-based functional activation, with older adults often showing increases in brain activation (e.g., greater bilateral prefrontal activation; Cabeza & Dennis, 2013; Davis et al., 2008; Diaz et al., 2021; Grady et al., 2015; Wierenga et al., 2008; Zhang et al., 2019). Although age-related differences in functional activation are commonly observed, the mechanism underlying such changes has been debated. Compensatory accounts suggest that age-related increases in functional activation may reflect greater neural engagement, which then translates to maintained or improved performance (Cabeza et al., 2018). On the other hand, dedifferentiation accounts suggest that increased activation reflects neural inefficiency and may lead to age-related declines in behavior (Li et al., 2001).

In addition to examining age-related differences in brain structure and task-based functional activation, another way to explore age-related differences in the brain is to investigate functional connectivity. Functional connectivity analyses use functional magnetic resonance imaging (fMRI) data to examine how signals from different brain regions covary (i.e., patterns of correlated brain activity). These patterns of functional connectivity reflect how different brain regions work together and interact with each other (Friston, 1994). When brain regions work together or are functionally connected, they are said to form networks. Common functional networks include the default mode network (DMN), the salience network (SN), and the frontal-parietal network (FP; Power et al., 2011), with each network serving different cognitive functions (e.g., DMN and monitoring, FP and attentional control). Moreover, functional connectivity analyses can be particularly useful in examining age-related differences as the approach minimizes task demands which can affect older and younger adults differentially. Data are often collected during rest where participants are asked to simply relax and look at a fixation cross. These resting state data reflect spontaneous brain activity in the absence of any explicit task.

Resting state data can be used to examine connectivity within a single network (i.e., within network connectivity), which reflects how activity is correlated among those regions and is thought to reflect network integrity. Many previous studies on age-related differences in brain activity have focused on specific networks such as the DMN or FP (Power et al., 2011). A common finding is that increased age is often associated with lower within-network functional connectivity (Betzel et al., 2014; Cao et al., 2014; Geerligs et al., 2015; Onoda et al., 2012; Siman-Tov et al., 2017; Song et al., 2014; Tomasi & Volkow, 2012; Zhang, Gertel, et al., 2021). Moreover, these age-related differences in within-network connectivity have been associated with worse behavioral performance across several aspects of cognition, including episodic memory (Wang et al., 2010), fluid intelligence (Onoda et al., 2012), and other cognitive abilities (for a review, see Sala-Llonch et al., 2015). Moreover, several studies have investigated the relationships between aging, functional connectivity, and language (Antonenko et al., 2013; Ferré et al., 2019; Gertel et al., 2020; Krieger-Redwood et al., 2019; Pistono et al., 2021; Zhang, Bai, et al., 2021). Consistent with the broader literature, studies that have examined within-network connectivity and language have found weaker within-network connectivity among older adults compared to younger adults, and stronger within-network connectivity has been associated with better language functions in, for example, semantic control (Wawrzyniak et al., 2017), verbal fluency (Miró-Padilla et al., 2017), and syntax (Antonenko et al., 2013).

While within-network activity reflects coherence or integrity of that network, networks often interact with one another both beneficially (e.g., coordinating the observation of an event with action) and detrimentally (e.g., failing to monitor because of distraction from a salient event). Thus, examining connectivity across networks is also important in understanding overall brain functioning. Between-network connectivity can be examined in two primary ways. The first approach considers all or most of the brain’s networks, and examines how networks interact with one another to provide an overall picture of network interaction and brain function (i.e., whole brain between-network functional connectivity). Previous studies taking this approach have found higher whole brain connectivity among older adults during language tasks such as synonym/antonym decisions and picture naming (Ferré et al., 2019). Examining the relationship of these findings to behavior revealed that higher whole brain between-network resting state connectivity was associated with poorer language ability among older adults (Zhang, Gertel, et al., 2021), suggesting that increased whole brain between-network connectivity is related to worse cognitive performance. The second approach is more focal and examines interactions between specific regions and/or networks to better understand how two networks or regions might interact. Studies that have taken this more focal approach suggest that some increases in connectivity may be compensatory (Gertel et al., 2020; Pistono et al., 2021). Gertel and colleagues found that although older adults had overall decreased functional connectivity compared to younger adults, older adults who had stronger resting state functional connectivity between left inferior frontal gyrus and right hemisphere executive function regions performed better on the Stroop task. These findings suggest that the increased RSFC between a core language region and executive function regions had a compensatory function. Similarly, Pistono et al. (2021) reported that stronger connections between the language network (i.e., a left lateralized frontal-temporal network involved in core language functions) and the multiple demand network (i.e., a bilateral frontal-parietal network involved in domain general cognitive functions) were associated with better verbal fluency performance in older adults. Thus, these two approaches to examining between-network connectivity provide slightly different views of brain function. The whole brain approach reflects overall brain function and has generally shown higher between-network connectivity for older adults that is associated with worse performance. The more focal approach suggests that higher between-network connectivity, at least between language and executive regions, may have a compensatory function (e.g., Cabeza et al., 2018; Reuter-Lorenz & Cappell, 2008).

However, examining either within-network or between-network connectivities in isolation does not illustrate the whole picture of aging and brain function. One way to integrate both within- and between-network characteristics is to use a measurement of network segregation (Chan et al., 2014). Network segregation compares the difference between within- and between-network connectivities divided by within-network connectivity. Thus, network segregation provides a more complete picture regarding the network organization across the whole brain. Several studies have used network segregation to investigate how it relates to age-related differences in cognition (Chan et al., 2014; Chan et al., 2018; King et al., 2018; Varangis et al., 2019). The findings showed that increasing age was associated with lower network segregation, consistent with previous findings looking at within- and between-network connectivity separately. Additionally, lower network segregation across the whole brain has been related to lower cognitive performance in the domains of speed, episodic memory, and fluid intelligence (Chan et al., 2014; Chan et al., 2018; King et al., 2018; Varangis et al., 2019). However, these integrated measures of brain function have not been used to examine language and aging to date.

Therefore, in the current study, we used a whole brain network approach to investigate the relationship between functional connectivity and age-related differences in language production. First, we used network segregation as our measure to examine how language production is related to language network integrity. However, language production involves more than just language ability per se; it also relies on perceptual regions for visual processes and object recognition and motor processing for articulation (Guenther et al., 2006; Tremblay et al., 2016), as well as other cognitive abilities such as monitoring, planning, and executive functioning (for review, see Burke & Shafto, 2008; Diaz et al., 2016). That is, language production ability may involve not only the language network, but also other brain networks. Therefore our second goal was to investigate how language production is associated with overall, whole brain network integrity, using network segregation calculated across the brain. Based on previous studies, we predicted that there would be main effects of age on both language production ability and network measures. Specifically, older adults would show lower language production ability and lower network segregation for both the language network and the whole brain. Additionally, we hypothesized that both network measures would be associated with language production ability and that more segregated networks would be related to better language production. Moreover, the patterns in these relationships can inform our understanding of cognitive aging. If increased age is related to weaker brain–behavior relationships, this would be consistent with a dedifferentiation account of aging (Ghisletta & Lindenberger, 2003; Li et al., 2001). On the other hand, if high performing older adults rely on a broader neural network to support language functions compared to younger adults, this would be consistent with compensatory accounts of aging (Cabeza et al., 2018).

## MATERIALS AND METHODS

### Participants

Ninety adults (ages: 22–78 yr) participated in the experiment. All participants were community-dwelling, right-handed, native English speakers who were not fluent in a second language. All participants had normal or corrected-to-normal vision and reported no history of neurological, psychological, or major medical conditions (Christensen et al., 1992). Eight participants were removed from the analysis because of missing data points—seven did not have picture naming data (5 did not finish the task and there were 2 recording failures) and one participant did not complete the story elicitation task. Three more participants were removed because of having outlier data points on behavioral measurements (see Data Analyses for details), leaving 79 participants’ data in the final analyses (ages: 22–78 yr, mean age = 48.2 yr, *SD* = 16.1 yr; 49 female).

Every participant first completed a behavioral testing session with a battery of psychometric and neuropsychological tests to assess basic cognitive functions such as speed, executive function, memory, and language. The psychometric tasks included the Mini-Mental State Exam to screen for mild cognitive impairment or dementia (MMSE; Folstein et al., 1975), and the Geriatric Depression Score (GDS) short version to screen for depression (Guerin et al., 2018; Sheikh & Yesavage, 1986). Participants also completed several standardized or adapted neuropsychological assessments. For processing speed and general executive function, participants completed simple (i.e., respond to a black square as quickly as possible) and choice (i.e., identify the direction of left/right arrows as quickly as possible) reaction time tests; WAIS-III vocabulary assessed vocabulary size, forward and backward digit span assessed working memory, and a computerized adaptation of the digit-symbol subtest assessed processing speed (Wechsler, 1997); and a computerized color Stroop task assessed executive function (i.e., make a response to the color of the ink when it is consistent/inconsisent with the word meaning (MacLeod, 1991; Stroop, 1935). Participants also completed a reading span task to assess verbal working memory (Conway et al., 2005), and the California Verbal Learning Test to assess immediate and delayed memory (i.e., one learning trial, 16 word list in 4 categories, one immediate recall assessment, one delayed recall assessment; Woods et al., 2006). For language assessments, participants completed the author recognition test and a comparative reading habit questionnaire to assess reading habits (Acheson et al., 2008).

Additionally, participants performed several tasks measuring different aspects of language production. These tasks included a verbal fluency task (Patterson, 2011), a picture naming task, and a speech elicitation task. During the verbal fluency task, participants were asked to overtly generate as many words as possible in each phonemic (F, A, S) and categorical (animals, supermarkets) category. Participants were given one minute per category to respond and were asked to avoid saying proper names of people or places, and repetitions of words. During the picture naming task, pictures of different objects were presented in the center of the screen and participants were asked to overtly name each picture as quickly and accurately as possible. During the speech elicitation task, participants were asked to generate a story from the picture book *Frog, Where Are You?*, by Mercer Mayer. They were encouraged to tell the story to the experimenter as if the experimenter had never heard the story before. Participants’ responses were recorded for offline analyses and there was no time limit for them to respond.

Demographic characteristics and assessment scores are reported in Table 1. All participants gave written, informed consent, and were paid for their participation ($15–30/hr). All procedures were approved by the Institutional Review Board at Pennsylvania State University.

**Table 1.** Participant demographics, neuropsychological testing scores, and correlation with age

Demographic information	Mean (*SD*)	Range	Age regression
*N*	79		
Age (years)	48.2 (16.1)	22–78	
Gender (M/F)	30/49		
Participant characteristics			
Education (years)	16.8 (3.1)	6–25	−0.009
MMSE (score out of 30)	28.8 (1.1)	19–30	−0.004
Depression (GDS) (score out of 15)	0.8 (1.0)	0–5	−0.01
Cognitive assessments			
Simple RT (box, ms)	298.1 (52.7)	237.3–479.9	0.78*
Choice RT (arrow, ms)	352.0 (75.6)	252.6–781.9	2.48***
WAIS vocabulary (score out of 66)	54.7 (5.2)	41–66	0.04
Digit symbol RT (ms)	1574.0 (384.6)	889.0–3001.0	16.5***
Digit span forward (score out of 16)	11.0 (2.1)	6–16	−0.003
Digit span backward (score out of 16)	7.3 (2.0)	4–14	−0.007
Stroop effect RT (Incongruent–Congruent, ms)	58.8 (80.6)	−63.7–420.0	1.90***
Verbal working memory (score out of 1)	0.4 (0.2)	0.02–0.8	−0.002
Immediate recall (score out of 16)	10.6 (2.5)	3–16	−0.05**
Delayed recall (score out of 16)	8.9 (2.9)	2–16	−0.05*
Author Recognition Test^a^ (score out of 76)	24.9 (14.1)	3–64	0.50***
Comparative reading (score out of 35)	26.0 (4.7)	11–35	0.05
Language production measures			
Verbal fluency (number of correct responses)	88.1 (14.9)	50–125	−0.24*
Phonemic fluency (F, A, S)	41.3 (9.5)	16–68	−0.06
Category fluency (animal and supermarket)	46.8 (8.7)	27–69	−0.18**
Picture naming RT (ms)	1046.7 (134.2)	741.3–1430.0	1.24
Story elicitation task			
Mean length of utterance	9.4 (2.1)	5.6–18.7	−0.01
Moving average type token ratio^b^	0.66 (0.04)	0.54–0.74	−0.0004

*Note*. The second column displays raw score means, with standard deviations (*SD*). The third column indicates the score range of each test. The fourth column indicates its regression coefficient with age. GDS = Geriatric Depression Score; MMSE = Mini-Mental State Exam; RT = reaction time; WAIS = WAIS-III: Wechsler Adult Intelligence Scale.

^a^ Author Recognition Test scores are calculated as the number of correct identifications − the number of incorrect responses.

^b^ MATTR used a moving window of 50 words.

* *p* < 0.05; ** *p* < 0.01; *** *p* < 0.001.

### Acquisition of MRI Data

All imaging data were acquired on a 3T Siemens Prisma Fit scanner using a 64-channel head coil. Localizer images were collected and used to define a volume for data collection, higher-order shimming, and alignment to the anterior commissure and posterior commissure (AC-PC). T1-weighted anatomical images were then collected using a magnetization-prepared rapid acquisition gradient echo (MPRAGE) sequence (repetition time [TR] = 2,300 ms; echo time [TE] = 2.28 ms; inversion time [TI] = 900 ms; flip angle = 8°; echo spacing = 7 ms; acceleration factor = 2; field of view [FOV] = 256 mm^2^; voxel size = 1 × 1 × 1 mm; 160 contiguous slices).

After the structural scan, blood-oxygen level dependent (BOLD) resting state data were acquired using an echoplanar imaging (EPI) sequence (TR = 2,000 ms; TE = 25.0 ms; flip angle = 90°; echo spacing = 0.49 ms; FOV = 240 mm^2^; voxel size = 3 × 3 × 4 mm; 33 contiguous slices, parallel to the AC-PC; phase encoding = anterior to posterior, fat saturation = on; slice acquisition = sequential, descending; volumes = 180; run duration = ∼6 min). Two additional volumes were acquired and deleted at the start of the scan to reach steady state equilibrium. During the resting state run, participants were instructed to relax in the scanner with their eyes open and to look at a fixation cross presented in the center of the screen without falling asleep. Four task-based runs using the same parameters as the resting state run were also collected after the resting state run (task run duration = ∼5.6 minutes). During the task runs, participants were presented with words and were asked to read aloud words as quickly and accurately as possible. Results from the task will be reported elsewhere; here we focus only on the resting state data.

Finally, a field map sequence was performed with a double-echo spoiled gradient echo sequence (TR = 446 ms; TE = 4.92 ms; flip angle = 60°; FOV = 240 mm^2^; voxel size = 3 × 3 × 4 mm; 33 contiguous slices; phase encoding = anterior to posterior, fat saturation = off; duration = 1:12 min) that generated two magnitude images and one phase image that were used for correcting susceptibility distortions in the functional data.

### fMRI Data Preprocessing

Data quality was first assessed using the fBIRN QA tool (Glover et al., 2012; https://www.nitrc.org/projects/bxh_xcede_tools/), measuring the number of potentially clipped voxels, mean signal fluctuation-to-noise ratio (SFNR), and per-slice variation. Additionally, the anatomical and functional images were visually inspected for artifacts and signal drop-out. Preprocessing analyses were carried out using the CONN functional connectivity toolbox (Version 18.a) under the MATLAB environment (Whitfield-Gabrieli & Nieto-Castanon, 2012). Preprocessing steps included functional realignment and unwarping to estimate and correct for participant motion, distortion correction using a voxel-displacement map calculated based on the field map, and a slice-timing correction which corrected for maturation of the BOLD signal over time (Huettel et al., 2004). Additionally, functional outliers were detected with an ART (Artifact Detection Tools)-based identification method (NITRC, 2015), in which outliers were defined using a conservative threshold (i.e., 97th percentile), and subsequently removed. All anatomical and functional images were normalized into standard Montreal Neurological Institute (MNI) space. The anatomical images were segmented into gray matter, white matter, and cerebral spinal fluid (CSF) tissue classes using SPM12 unified segmentation and normalization procedure, then these masks were applied to the functional images (Ashburner & Friston, 2005). During registration, functional images were aligned to anatomical images and both were normalized to standard space. A smoothing kernel of 6 mm was used to increase the signal-to-noise ratio, as well as to reduce spurious activations of single voxels. During denoising, the representative noise signal from white matter (5 components) and CSF (5 components) was extracted, and any signal correlated with these components was removed from the BOLD signal. The noise removal used the CompCor approach, which extracts multiple signals from CSF and white matter areas to capture motion and physiological artifacts while excluding neural signals, which avoids introducing artifactual negative correlations in the connectivity measures (Chai et al., 2012; Liu et al., 2017; Liu et al., 2021). To eliminate frequencies of less interest, a band-pass filter (0.008, 0.09) was used (Davey et al., 2013; Gohel & Biswal, 2015; Hallquist et al., 2013). The effects of the following quality assurance parameters were controlled for during data analysis: number of outlier and non-outlier scans (outlier threshold = 0.5 mm), max and mean motion, and max and mean global BOLD signal changes (outlier threshold = global-signal *z*-value of 3). The average number of invalid scans was 1.9 out of 180 scans/volumes (1%, *SD* = 5.4), and it was not significantly affected by age (*β* = 0.71, *SE* = 0.60, *p* = 0.24). The mean amount of motion was 0.20 mm (*SD* = 0.08 mm), and increased age was associated with higher head motion (*β* = 0.03, *SE* = 0.01, *p* < 0.001). The analyses removing variance associated with the variables described above occurred in a single linear regression step, and the residualized BOLD signal was used for further statistical analyses.

### Node Definition and Network Measures

Power et al. (2011) identified 264 coordinates in the brain and created 5 mm fixed-radius sphere nodes around these locations. We also used the same 264 locations and created the 5 mm radius non-overlapping nodes using the MNI152, 2 mm brain as the reference. Power identified 12 networks (hand somatomotor, mouth somatomotor, visual, salience, auditory, cingulo-opercular control, frontoparietal control, ventral attention, dorsal attention, default, subcortical, and cerebellar) and divided nodes into these networks. Among all nodes, 33 were excluded from the analysis due to poor classification fit with the Power networks. To further identify nodes that belong to the language network, we used the language regions identified by Fedorenko et al. (2010; left language network and right hemisphere homologues). We selected the networks defined by Fedorenko and colleagues because the regions were identified functionally (sentences > nonwords), were replicable within subjects, had a clear correspondence across subjects and support broad language processes. Although it can be argued that this network is a comprehension-based network, follow-up studies also suggested that it overlaps significantly with the production network (Hu et al., 2023). The language network supports both lexical access and sentence generation during language production. Therefore, the left language network they defined represents a broad language processing network that supports both language comprehension and production. After defining these left language and right hemisphere homologue networks, any nodes that overlapped with these two localizers were categorized as the left language network and the right hemisphere homologue network. The remaining nodes were then binned across the 12 Power networks according to their location. Nodes were double checked to ensure that no location belonged to more than one network. The final set included 231 nonoverlapping nodes belonging to 14 networks. (See Table S1 in the Supporting Information, available at https://doi.org/10.1162/nol_a_00106, for MNI coordinates for node locations.)

For each participant, the resting state time series of each node was extracted, then a cross-correlation of each node’s time course with every other node’s time course was calculated. This was performed using the CONN functional connectivity toolbox (Version 18.a) under the MATLAB environment (Whitfield-Gabrieli & Nieto-Castanon, 2012). Correlation coefficients were converted to *Z*-values using Fisher’s equation. Consistent with previous studies using similar approaches (Chan et al., 2014), negative correlations were not included in further analysis because of uncertainty regarding the meaning of negative correlations (Hallquist & Hillary, 2018). The final matrix for each participant was a 231 × 231 weighted *Z*-matrix with the diagonal and negative values set to zero. Correlation matrices were then imported in R for further processing (RStudio Team, 2022). The following R packages were used in the analyses: readxl (Wickham & Bryan, 2019); tidyverse (Wickham et al., 2019); car (Fox & Weisberg, 2019); interactions (Long, 2019); ggplot2 (Wickham, 2016); and ggpubr (Kassambara, 2020).

Using the same methods as Chan et al. (2014), for each participant we calculated network segregation for the left language network and across the whole brain. First, network definitions were applied to each participant’s 231 × 231 weighted correlation matrix comprising 14 networks. Then, for each network, within-network connectivity was calculated as the mean node-to-node correlation of all nodes in that network (i.e., diagonal blocks). Between-network connectivity was calculated as the mean correlation value between each node in one network and the rest of the nodes outside of that network (i.e., off diagonal blocks). Finally, network segregation was calculated as the difference between within-network connectivity and between-network connectivity for a specific network divided by the within-network connectivity of that network. The overall, whole brain network measures were calculated by averaging the network segregation measures across all networks. All of the measures were calculated at the subject level first before calculating group-level results (i.e., each subject had one value for left language network segregation, and one value for overall network segregation).

### Data Analyses

As mentioned earlier, participants performed a series of neuropsychological tests to measure cognitive functions across different domains (see Table 1 for the list of cognitive assessments). For the purpose of the current study, we focused on variables measuring different aspects of spoken language production (i.e., verbal fluency, picture naming, and story elicitation). For verbal fluency, repetitions of the same words, incorrect responses, and proper names were excluded from the analysis. Responses were included if they matched the cue, and mythical animals were counted as correct for the animals category (e.g., unicorn, hippogriff). The total number of included responses for all categories were used as the verbal fluency score. For the picture naming task, participants’ responses were coded offline and only responses that matched the picture were coded as correct. The mean reaction time for correctly named pictures was used as the picture naming performance score. For the story elicitation task, participants’ stories were first transcribed using the CLAN software (MacWhinney & Wagner, 2010). For each participant, the mean length of utterances (MLU) measures utterance length by calculating the ratio of total morphemes to the number of utterances, representing speech complexity. An utterance is defined as a string of words that is followed by a pause of one second or more, ends with a terminal intonation contour, or has a complete grammatical structure (not a necessary feature). Moving average type token ratio (MATTR) can capture lexical diversity better than the traditional TTR measurement. TTR is obtained by dividing the number of different words by the total number of words in the text, which is constrained by the overall document length. MATTR, on the other hand, uses a fixed window length (50 words in the current study) and computes the TTR for a moving window (1–50, 2–51, and so on to the end of the text), and then the mean TTR is calculated for all windows. Therefore, MATTR is a length-invariant measurement of lexical diversity of spoken language production. All coding was performed by trained research assistants and verified by a second research assistant.

To reflect the overall language production ability and different aspects of speech (e.g., speed, complexity, and lexical diversity), a composite score was calculated for each participant by adding up the *Z* scores of the four production measurements (verbal fluency total, picture naming reaction time, MLU, and MATTR; Table 1). To make the direction of effects consistent across variables (i.e., higher values reflecting better ability), picture naming reaction times were reverse coded. To investigate age-related differences in language production, a linear regression was conducted on the language production composite scores, including age as the predictor. Three outliers were identified and removed from all analyses (Cook’s Distance greater than 4/sample size; Cook, 1979). Therefore, all analyses included a final set of 79 participants. Finally, all independent variables were standardized using the scale function in the R environment ((score-mean)/*SD*) prior to conducting the linear regressions described below.

To investigate the relationships among age, brain network characteristics, and language production, we first looked at age-related differences in language production by conducting a linear regression of age on the language production composite scores. Next, because we were interested in the language network, a simple linear regression was conducted on network segregation within the left language network, including age as the predictor. To look at the overall, whole brain structure, we also fit a linear regression on the whole brain network segregation while including age as the predictor. Furthermore, to investigate how language production relates to the language network segregation as well as the whole brain network structure, we conducted two sets of analyses on the language production composite scores. One set looked at the contribution of the left language network to language production, and included age, left language network segregation, and their interaction as independent variables. The other set of analyses explored the contribution of the whole brain network structure to language production, and independent variables included the main effects of age and whole brain network segregation, as well as their interaction. For cases where the interactions were significant, Johnson-Neyman tests were then conducted to identify the age ranges where the relationships between network measures and the language production composite scores were significant (Esarey & Sumner, 2018; Johnson & Fay, 1950). Specifically, the Johnson-Neyman test reports the value or values of the moderator (i.e., age) at which the effect of the predictor (i.e., network measures) on the dependent variable (i.e., language production composite scores) was significant. Additionally, given that head motion significantly differed across age, as indicated in the fMRI Data Preprocessing section, all network analyses included the amount of motion as a control variable to account for any remaining confounding effects of motion.

Finally, although we focused on the relationships between a broad language production measure and whole brain network segregation, we conducted additional analyses on each language production variable and the relationships between the language network and each other network to provide a more comprehensive picture. These analyses are touched upon briefly in the results and reported in full in the Supporting Information.

## RESULTS

### Age-Related Differences in Language Production

**Figure 1.**
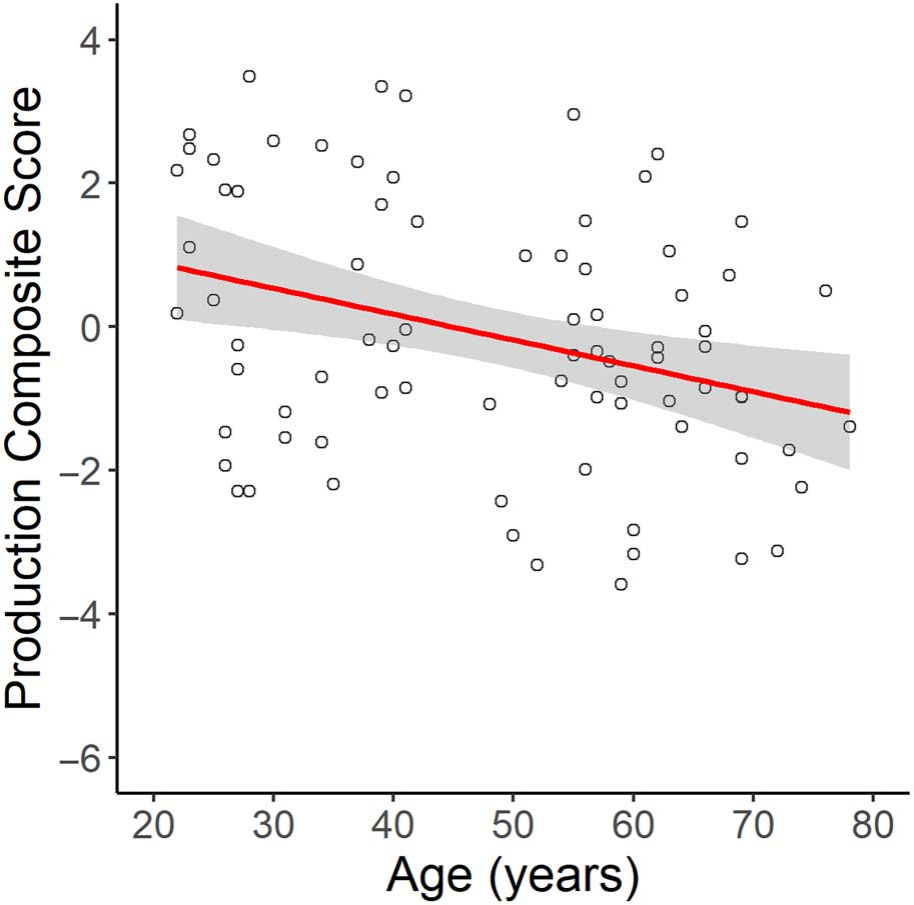
The main effect of age on the language production composite score. Age was significantly negatively correlated with production scores, where lower production scores indicate worse performance.

To examine age-related differences in language production, we conducted a linear regression of age on the production composite scores. This showed that there was a significant effect of age on language production (*β* = −0.59, *SE* = 0.20, *p* = 0.003). Specifically, increasing age was associated with lower production scores, indicating lower language production ability (Figure 1). As shown in Table 1 and Figure S2 in the Supporting Information, when looking at the age effects on each language production measurement separately, there was only a significant effect of age on the verbal fluency score (*β* = −0.24, *SE* = 0.12, *p* = 0.04).

### Age-Related Differences in Network Structure

**Figure 2.**
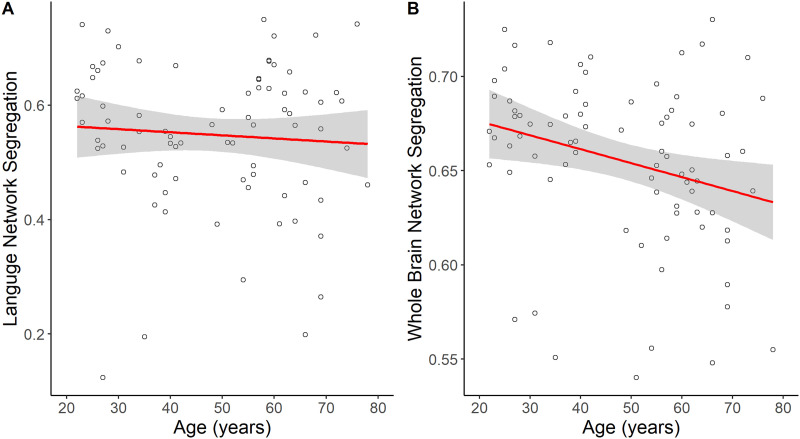
The main effects of age on language network segregation and whole brain network segregation. The effect of age on (A) language network segregation was not significant, but (B) whole brain network segregation was significantly lower with increasing age.

Linear regressions were conducted to investigate the effects of age on network segregation in the language network and across the whole brain. Although there was no significant relationship between age and left language network segregation (*β* = −0.01, *SE* = 0.02, *p* = 0.47; Figure 2A), older age was significantly associated with lower whole brain network segregation (*β* = −0.01, *SE* = 0.01, *p* = 0.02; Figure 2B).

### Relationship Among Age, Network Structure, and Language Production

**Figure 3.**
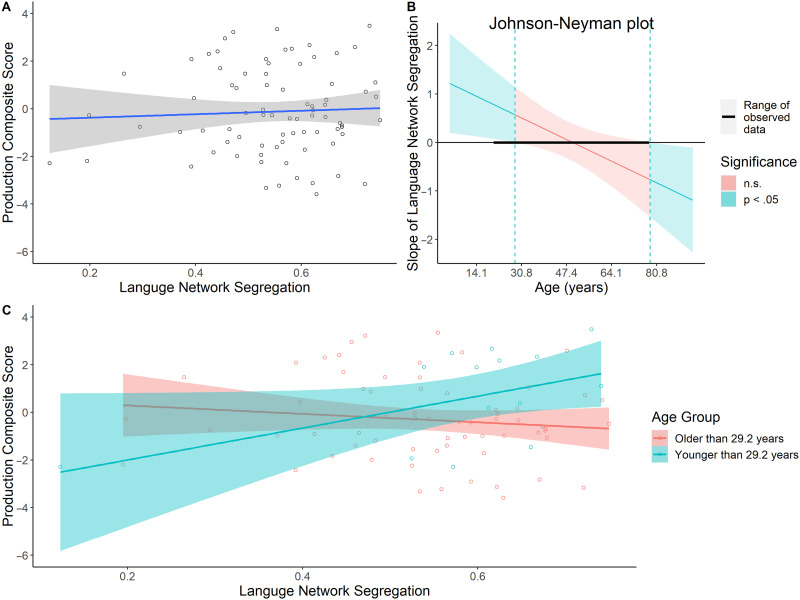
Age-related differences in the relationship between language network segregation and language production composite scores. (A) There was no significant relationship between language network segregation and language production. (B) The age ranges for which the Johnson-Neyman test identified a significant interaction between language network segregation and language production ability (22–29.2 yr old, for our sample). (C) Although there was no significant main effect of language network segregation on language production, the interaction between these variables was significant. For younger adults (< 29.2 yr old), there was a significant positive relationship between language network segregation and language production scores. This relationship was not significant in adults older than 29.2 years old. n.s. = not signitficant.

To look at how language production ability relates to the language network as well as the whole brain network, we conducted two analyses, one using network segregation of the language network and one examining whole brain network segregation. For the first analysis, a regression was conducted on the language production composite scores, including age, left language network segregation, and their interaction as independent variables. Consistent with the results from our previous regression, increasing age was significantly associated with lower language production composite scores (*β* = −0.55, *SE* = 0.21, *p* = 0.01). Although the main effect of language network segregation on the language production composite scores was not significant (*β* = 0.06, *SE* = 0.19, *p* = 0.75; Figure 3A), the interaction between age and language network segregation was significant (*β* = −0.45, *SE* = 0.18, *p* = 0.02). To further specify the interaction, we conducted a Johnson-Neyman test (Figure 3B). Results showed that the positive relationship between language network segregation and language production scores was significant at ages up to 29.2 years. These results suggest that higher language network segregation was associated with better language production ability but only in relatively young adults (Figure 3C).

**Figure 4.**
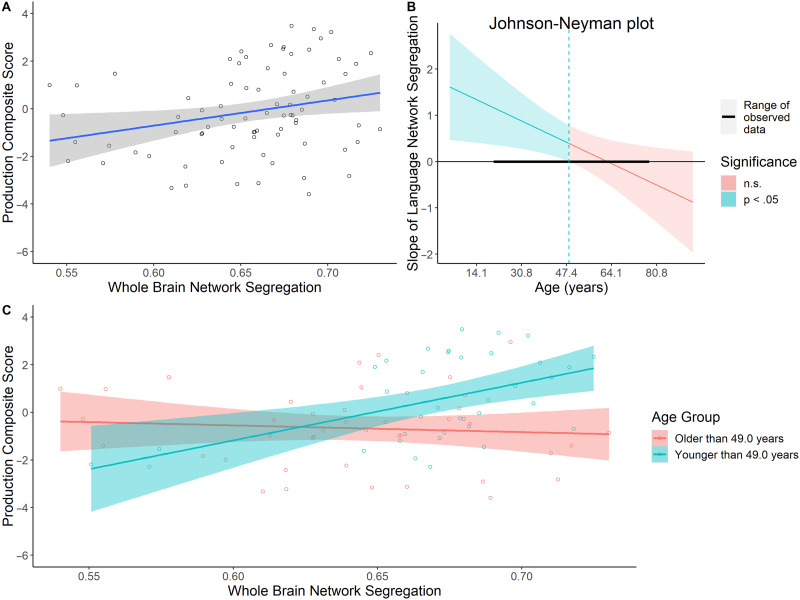
Age-related differences in the relationship between whole brain network segregation and language production composite scores. (A) There was a significant positive relationship between whole brain network segregation and language production. (B) The Johnson-Neyman test identified that the interaction was significant for ages 22–49.0 in our sample. (C) The significant main effect and interaction between whole brain network segregation and language production was driven by adults younger than 49.0 years old. This relationship was not significant in adults older than 49.0 years old.

Second, we investigated how language production was affected by the whole brain network structure and age using a regression on the language production composite scores while including age, whole brain network segregation, and their interaction in the model. Consistent with prior models, age was significantly negatively associated with language production composite scores (*β* = −0.44, *SE* = 0.21, *p* = 0.04). The main effect of whole brain network segregation on the language production composite scores was significant (*β* = 0.42, *SE* = 0.20, *p* = 0.04). Specifically, higher whole brain network segregation was associated with higher production scores in general (Figure 4A). Moreover, the interaction between age and the whole brain network segregation was significant (*β* = −0.46, *SE* = 0.19, *p* = 0.02). The Johnson-Neyman test indicated that for individuals younger than 49.0 years of age, there was a significant relationship between whole brain network segregation and language production scores, with higher network segregation associated with higher language production scores. This suggests that having more segregated networks in general was associated with better language production in younger and middle-aged adults (Figure 4B and 4C).

Moreover, since older age was significantly associated with lower gray matter volume (*β* = −0.80, *SE* = 0.07, *p* < 0.001), one potential concern is that the relationships among age, network segregation, and language production were confounded with age-related differences in gray matter volume. To address this concern, we re-ran all analyses including gray matter volume as a control variable. Results showed that the interaction between age and language network segregation (*β* = −0.41, *SE* = 0.19, *p* = 0.03), and the interaction between age and whole brain network segregation (*β* = −0.44, *SE* = 0.20, *p* = 0.03) on language production were both still significant. Lastly, further analyses on each language production variable separately, which is reported in full in the Supporting Information, showed that only picture naming speed was marginally predicted by interactions between age and network measures (with language network segregation, *β* = −28.89, *SE* = 15.47, *p* = 0.07; with whole brain network segregation, *β* = −30.18, *SE* = 16.82, *p* = 0.08).

## DISCUSSION

Older adults often exhibit declines in spoken language production such as slower speech rates (Duchin & Mysak, 1987; Mortensen et al., 2006; Spieler & Griffin, 2006), increased word finding difficulties, increased pauses and fillers in speech (Burke & Shafto, 2008; Horton et al., 2010; MacKay & James, 2004), and more dysfluent and less grammatically complex speech (e.g., Bortfeld et al., 2001; Kemper et al., 2003; Obler & Albert, 1981). Studies have suggested that these age-related differences in language production may be related to declines in phonological processing (Burke et al., 1991; Burke & Shafto, 2008), aspects of executive function (Hasher et al., 1991; Hoffman et al., 2018; Lustig et al., 2007), or processing speed (Salthouse, 1996, 2000). Despite the abundant discussion regarding potential behavioral mechanisms, the neural bases underlying these age-related differences are not entirely clear. Moreover, there is general disagreement in the aging literature about how commonly observed age-related neural differences should be interpreted, that is, as compensatory (Cabeza et al., 2018) or neural dedifferentiation (Li et al., 2001). Specific to neural activities underlying language production, some task-based fMRI studies have reported patterns supporting compensation (e.g., additional brain activation associated with maintained behavioral performance; Nagels et al., 2012; Wierenga et al., 2008) while others have found evidence for dedifferentiation (e.g., increased brain activation related to weakened behavioral performance; Diaz et al., 2014; Meinzer et al., 2009). In the current study, we focused on resting state functional connectivity and investigated how the network characteristics in the language network and across the whole brain relate to language production across adulthood. We specifically focused on a network measure called network segregation, which incorporates both within-network connectivity and between-network connectivity and highlights the degree to which networks are differentiated. We hypothesized that language production performance, functional connectivity, and their relationship would differ as a function of age.

As predicted, behaviorally, we found that older adults showed worse performance in language production tasks. This is consistent with previous studies that have reported age-related declines in production (for review, see Burke & Shafto, 2008; Diaz et al., 2016). This study, however, enhances previous findings by demonstrating this main effect of age on a composite score of language production from several different tasks, reflecting an overall profile of language production in a broad sample of adults (ages 22–78 yr). Although we focused on an integrated measure of language production ability based on multiple tasks, we also analyzed the effect of age on each production task performance separately (verbal fluency total score, picture naming speed, MLU, MATTR; reported in Supporting Information). Briefly, although all production measures showed the same decreasing trend with age, the effect of age was only significant on verbal fluency total score. The age effect on verbal fluency has been commonly observed (Gonzalez-Burgos et al., 2019; Rodríguez-Aranda & Martinussen, 2006; Troyer, 2000; Troyer et al., 1998). Compared to other measures, verbal fluency performance relies on a broader set of abilities reflecting language production, vocabulary, speed, as well as executive function. Therefore, the age-related difference in verbal fluency scores is likely influenced by age-related differences in general cognitive functions as well as language. In short, we found that older age was associated with worse overall language production, which might be driven by the age effect on lexical access and executive function.

In addition to the behavioral results, our network analyses showed that while network segregation within the left hemisphere language network was maintained in adults of all ages, increased age was associated with lower whole brain network segregation. Moreover, we examined how these network segregation results related to language production ability. For both network segregation within the left hemisphere language network and whole brain network segregation, there were significant interactions with age, such that increases in network segregation were associated with better language production abilities, but only for younger and middle-aged adults. Interestingly, the whole brain network segregation contributed to language production ability over a broader age range (up to age 49 yr) compared to the language network segregation (up to age 29.2 yr). This could reflect the reliance of language production on other cognitive abilities and could also be influenced by the stability of the left hemisphere language network segregation in adulthood. Below, we examine these findings in more detail.

First, consistent with previous studies of network segregation, older age was associated with lower whole brain network segregation (Chan et al., 2014; Chan et al., 2018; Geerligs et al., 2015; Petrican et al., 2017). Network segregation was calculated based on both within- and between-network connectivities such that higher within-network connectivity and/or lower between-network connectivity could lead to higher network segregation. On the whole brain level, network segregation reflects the degree to which different networks in the brain share connections among one another, thus, indicating a change in system specialization. As people age, brain regions, especially those within the same networks, may show lower connectivity strength (see Figure S1 in the Supporting Information). These age-related differences may reflect a loss of functional specificity given that lower whole brain network segregation was associated with worse language production performance in the current study, and lower network segregation has also been associated with lower memory function in previous studies (Chan et al., 2014). Therefore, lower whole brain network segregation in older adults reflects a reduction in overall functional specificity of network-based processes, supporting the dedifferentiation account of aging (Ghisletta & Lindenberger, 2003). Despite significant age-related differences in the whole brain network segregation, there was no significant main effect of age on language network segregation. To our knowledge, no study has specifically looked at age differences in the resting state language network using the integrated network segregation measurement. The maintenance of the language network segregation across adulthood suggests that the functional specificity of the language network and its interactions with other networks is relatively stable across the lifespan. Although we focused on spoken language production in the current study, the stability in the language network is, in fact, consistent with behavioral findings showing that aspects of language comprehension, semantic processing, and vocabulary are well maintained and even improve with age (e.g., Balota & Duchek, 1988; Burke & Peters, 1986; Cohen-Shikora & Balota, 2016; Howard et al., 1981; Madden et al., 1993; Verhaeghen, 2003). These findings suggest that, unlike other types of cognitive functions or neural structures that are vulnerable to aging, many basic language functions, except speech, and the core neural structures related to language are relatively well preserved regardless of increasing age.

Moreover, language production was associated with network segregation and this relationship was modulated by age, even after controlling for the age-related differences in gray matter volume. Specifically, only in relatively younger adults, higher network segregation in both the language network and the whole brain network was associated with better language production. These results indicate that more differentiated resting state networks may contribute to enhanced language production ability in younger and middle-aged adults, consistent with previous studies using measures similar to network segregation (e.g., Duncan & Small, 2016). For instance, although focusing on individuals with aphasia, Duncan and Small (2016) also found that increased resting state network modularity (a measure similar to network segregation) was positively associated with better performance on a narrative task.

Yet, this positive relationship between network segregation and language production was not significant in neurotypical older adults. This result is consistent with previous studies that have examined the relationship between age-related differences in language function and resting state networks. For example, Antonenko et al. (2013) found that younger adults showed superior syntactic performance compared to older adults. Critically, only in younger, but not older adults, stronger connectivity within the language network was associated with enhanced behavioral performance. The lack of a brain–behavior relationship among older adults is also consistent with other studies on language and aging that focused on task-based functional activation. For example, Diaz et al. (2014) reported that younger adults exhibited significant correlations between fMRI activation and language function, which was not present in older adults. These age-related differences in brain–behavior relationships in general, support the dedifferentiation account of aging, which suggests that as people get older, their functional networks become less structured and the relationships between functional measures and behavior weaken (Ghisletta & Lindenberger, 2003).

At first glance, these findings supporting the dedifferentiation account seem to be inconsistent with some previous language studies that reported results supporting compensatory neural accounts of aging (Gertel et al., 2020; Pistono et al., 2021). In these studies, they found that increased functional connectivities between language nodes and other networks were associated with better language function in older adults. However, it is worthwhile to mention that these studies and the current study differ in several aspects. Foremost, the previous studies incorporated an extreme-groups design that included two distinct age groups and used age as a categorical variable, while the current study investigated the effects of age across adulthood and treated age as a continuous variable. Critically, earlier studies focused on local relationships between specific language nodes and domain-general regions, while the current study focused on system-wide structures (i.e., between the language network and the rest of the brain and across the whole brain). Specifically, rather than looking at a language network, Gertel et al. (2020) took a more focused approach and included only one language region (left inferior frontal gyrus) as the seed and found that the connectivity between this seed and the right hemisphere executive function regions led to better Stroop performance in older adults. Although the Stroop task involves language processing, it also requires a high degree of cognitive control. So, the strong relationship between a language seed and executive function regions was expected. Although Pistono et al. (2021) examined networks rather than individual regions, they focused on the local relationships between two networks—the language network and the multiple-demand network—and did not examine the rest of the networks in the brain. It could be the case that reorganization of individual regions or select networks can serve a beneficial function or reveal less age-related decline (i.e., the nonsignificant effect of age on language network segregation), while whole brain analyses show an overall pattern of dedifferetiation in aging.

There is another interesting observation regarding the relationships among network segregation, age, and language production. Specifically, the whole brain network segregation seems to contribute to language production to a greater extent than the language network segregation, as reflected by the fact that the relationship between the network segregation and production was significant through middle age whereas the relationship between language network segregation and production was only significant for younger adults (49 yr old for whole brain network segregation vs. 29.2 yr old for language network segregation). Recall that there was no significant main effect of age on language network segregation, suggesting that the core language network remains stable with age. Additionally, the main effect of language network segregation on language production was not significant. At first glance, these results may be surprising because one would expect that language function should largely rely on the language network. However, language production and age-related differences in language are multifaceted. For example, studies have shown that core language functions such as comprehension or vocabulary are largely intact across adulthood (Madden et al., 1993; Verhaeghen, 2003; Waters & Caplan, 2001). On the other hand, spoken language production often declines with age. According to behavioral hypotheses such as the inhibition deficit theory (Lustig et al., 2007) or the processing speed theory (Salthouse, 2000), age-related differences in production may be in part because it relies on other domains such as visual and motor processing, planning, monitoring, memory, and control, which suffer from age-related decline (for review, see Burke & Shafto, 2008; Diaz et al., 2016). Although a more thorough investigation is needed, a quick exploration of our data showed that enhanced production was associated with better performance on the digit symbol task (*β* = 0.57, *SE* = 0.20, *p* = 0.005), suggesting that language production covaries with domain general cognitive abilities. Therefore, while language comprehension may largely rely on the core language network (Wehbe et al., 2021), language production relies on not only the language network but also many other networks that support other cognitive functions (e.g., mouth somatomotor network for phonological production, auditory network for hearing, ventral and dorsal attention networks, frontal-parietal control networks). Finally, the age-related stability in the left hemisphere language network segregation may itself contribute to the weaker relationship between language network segregation and language production ability. That is, if there is reduced variability in one measure and higher variability in another, a significant relationship between the two will be less likely mathematically. The results of the present study suggest network segregation across a broader set of networks may be required to support superior language production performance.

To summarize, language network segregation was stable across adulthood, highlighting the neural stability of left hemisphere language regions and their interactions with other brain regions. However, we found that increased age was associated with worse language production performance and less segregated whole brain networks. Critically, whole brain network segregation was associated with enhanced language production, but only among younger and middle-aged adults. In contrast, older adults exhibited weaker relationships between network segregation and language production. These results are consistent with the dedifferentiation account of aging. Findings from the current study also indicate the contribution of the whole brain network to production, implying that the age-related differences in language production may be related to age-related differences in general cognitive abilities.

## ACKNOWLEDGMENTS

This project was funded by a National Institutes of Health (NIH) National Institute on Aging (NIA) grant. The writing of this paper was supported by the National Natural Science Foundation of China and by the Start-up Research Grant (SRG2022–00003-ICI) and Multi-Year Research Grant (MYRG2022–00148-ICI) from the University of Macau. We thank the staff and scientists at the Social, Life, and Engineering Sciences Imaging Center (SLEIC) and the Center for Language Science (CLS), where the study was conducted, for their support.

## FUNDING INFORMATION

Michele T. Diaz, National Institute on Aging (https://dx.doi.org/10.13039/100000049), Award ID: R01 AG034138. Haoyun Zhang, National Natural Science Foundation of China (https://dx.doi.org/10.13039/501100001809), Award ID: 32200845.

## AUTHOR CONTRIBUTIONS

**Haoyun Zhang**: Conceptualization, Formal analysis, Investigation, Methodology, Visualization, Writing – original draft. **Michele T. Diaz**: Conceptualization, Funding acquisition, Methodology, Project administration, Supervision, Writing – review & editing.

## DATA AND CODE AVAILABILITY STATEMENTS

Data, analysis scripts, and supplementary materials are available at https://osf.io/3u9rw/?view_only=4ba25920ed834408bda0fccd96f4cc2e.

## TECHNICAL TERMS

Resting state functional connectivity: A method that examines how brain regions work together and thus how they form functional networks without additional task demands.
Network segregation: A measure that compares the difference between within- and between-network connectivities divided by within-network connectivity, providing a more complete picture regarding the network organization across the whole brain.
