# Supplementary material for: Resting State Network Segregation Modulates Age-Related Differences in Language Production
Source: Neurobiol Lang (Camb). 2023 Jun 13;4(2):382–403. doi: 10.1162/nol_a_00106 (PMC10403275; doi:10.1162/nol_a_00106)
Supplement: Supplementary file 1 [file nol-4-2-382-s001.zip › supps/Supplementary_Materials_Dec29_2022.docx]

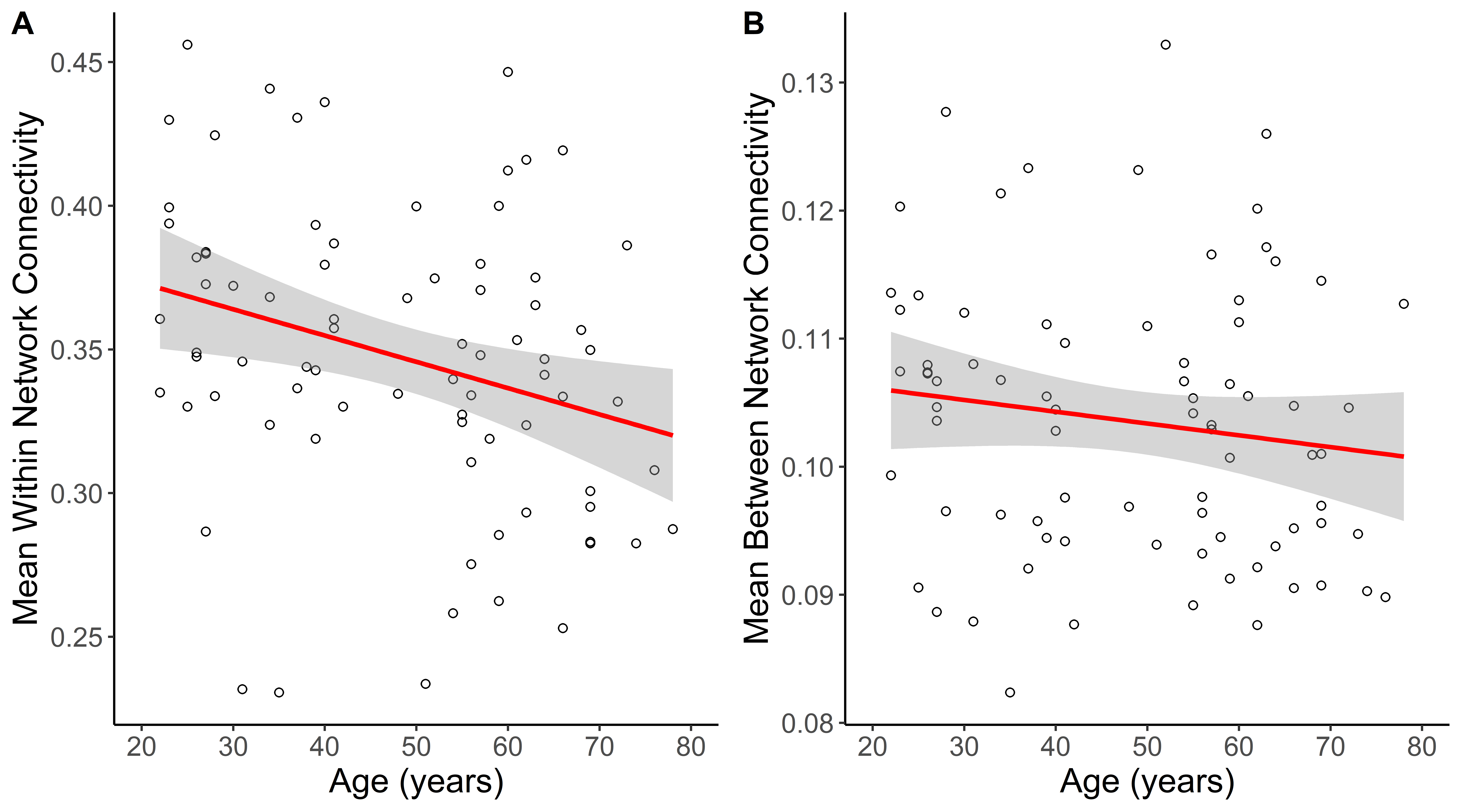


*Supplementary Figure 1. The main effects of age on A) whole brain mean within network connectivity, and B) whole brain mean between network connectivity. Older age was associated with lower mean within network connectivity, while there was no significant relationship between age and mean between network connectivity.*

**Analyses on Each Language Production Measurement**

Language production involves not only the lexical processing, but also many other aspects such as vocabulary knowledge, speech complexity, and processing speed. In the main manuscript, we used an integrated language production composite score reflecting a global spoken production ability. However, extracting distinct constructs from the language production tasks, and examining their relationship with network segregation and age could be more informative. Specifically, the verbal fluency total score measures production, executive function, and vocabulary, the MATTR reflects lexical diversity, the picture naming reaction time incorporates processing speed, and the MLU measures speech complexity. Therefore, in the supplementary materials, we investigated the effect of age, as well as its interaction with network measures on each language production measurement separately. Note that picture naming reaction time was reverse coded as picture naming speed so that the direction is consistent with other variables (i.e., higher values reflecting better ability). Given the exploratory nature of the analysis, we did not correct for multiple comparisons.

*Age-related Differences in Different Language Production Measures*

To examine age-related differences in different language production aspects, we conducted linear regressions of age on the different production variables. Results showed that increased age was only significantly associated with lower verbal fluency total score (*β* = -3.9, SE = 1.9, *p* = .04), while the effects of age were not significant on other variables (i.e., picture naming speed, MLU, MATTR; *p*s > .1). These results have been reported in Table 1 in the manuscript, and Supplementary Figure 2.





*Supplementary Figure 2. The main effect of age on different language variables. A) Age was significantly negatively correlated with verbal fluency total score, where lower production scores indicate worse performance. B), C), and D) showed that the effects of age were not significant on picture naming speed, MLU, or MATTR.*

*Relationship among Age, Network Structure, and Language Production*

To look at how different language production variables relate to network measures, we conducted two analyses (one with language network, and one with whole brain network segregation) on each language variable. Only on picture naming speed, there were marginally significant interactions between age and network measures (with language network segregation, *β* = -28.89, SE = 15.47, *p* = .07; with whole brain network segregation, *β* = -30.18, SE = 16.82, *p* = .08). While a Johnson-Neyman test could not identify a range of significance for non-significant interactions, we plotted these interaction effects using the age markers identified from the language composite score (i.e., 29.2 years for language network segregation, and 49 years for whole brain network segregation; Supplementary Figure 3). The plots showed that the effects of age and network segregation on picture naming speed were consistent with the effects on language production score, such that only in younger adults, there seems to be a relationship between network segregation and production. These results indicated that the relationships among age, network segregation, and language production composite scores were likely to be driven by the age differences in the more constrained production demands.


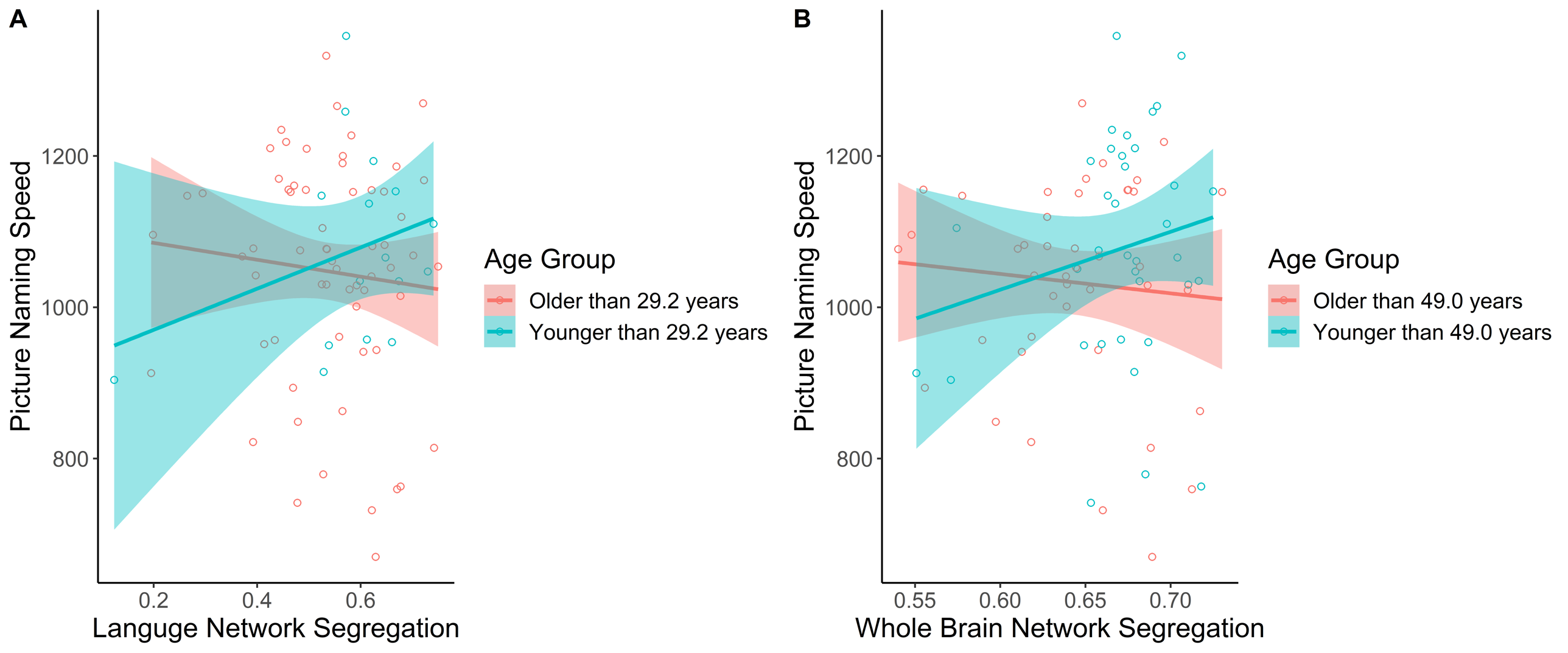


*Supplementary Figure 3. Age-related differences in the relationship between network segregation (A, language network; B, whole brain network) and language production composite scores. Consistent with the results with the language production composite score, only younger adults showed significant positive relationships between network segregation and picture naming speed. These relationships were not significant among older adults.*

**Analyses on the Relationship between Language Network and Other Networks**

The main analysis reported in the manuscript showed that there was a significant interaction between age and language network segregation on the language composite score, indicating that age modulated the brain-behavior relationship. The language network segregation was calculated based on the within language network connectivity, and its relationships with all the other networks. Yet, it is not clear, for example, which between network relationship is mostly related to production performance and modulated by age. We did follow up analyses to clarify these questions. Thirteen separate models were conducted (with Right language homologues, Hand somatomotor, Mouth somatomotor, Visual, Salience, Auditory, Cingulo-opercular control, Fronto-parietal control, Ventral attention, Dorsal attention, Default, Subcortical, and Cerebellar). Specifically, for each of these networks, linear regressions were conducted on language composite scores including age, within language network connectivity, its between network connectivity with the target network, and their interactions as predictors in the models. Using the fronto-parietal control network as an example, the linear regression on the language composite score included age, within language network connectivity, between language-fronto-parietal network connectivity, interaction between age and within language network connectivity, and the interaction between age and the between language-fronto-parietal network connectivity. Because of the exploratory nature of the analysis, we did not apply any multiple corrections.

Among all 13 analyses on the language production composite score, only the interactions between age and the fronto-parietal control network (*β* = 11.56, SE = 5.89, *p* = .05), and between age and the cerebellar network (*β* = 9.35, SE = 4.89, *p* = .06) were marginally significant. While a Johnson-Neyman test could not easily identify a range of significance for non-significant interactions, we plotted these interaction effects using the age markers identified from the relationships among language network segregation, age, and language composite score (i.e., 29.2 years, Supplementary Figure 4). Although the relationship between the production score and the between language and fronto-parietal control network connectivity was by and large flat for both age groups (Supplementary Figure 4A), the relationship between the composite production score and the between language and cerebellar network connectivity was modulated by age (Supplementary Figure 4B). Specifically, only in younger adults, weaker connectivity between the language network and the cerebellar network was associated with higher language production scores, consistent with the results reported in the main manuscript that stronger language network segregation (i.e., higher network integrity, caused by higher within and lower between network connectivities) in younger adults was associated with enhanced language production ability. These results indicated that the significant interaction between age and language network segregation on language production performance reported in the main manuscript seems to be driven mainly from the relationship with the cerebellar network, it also might be related to the relationship with the fronto-parietal network. Overall, a more segregated language network (high within network connectivity, and low between network connectivity) may suggest a more efficient network structure, and therefore, better support for language production functions.


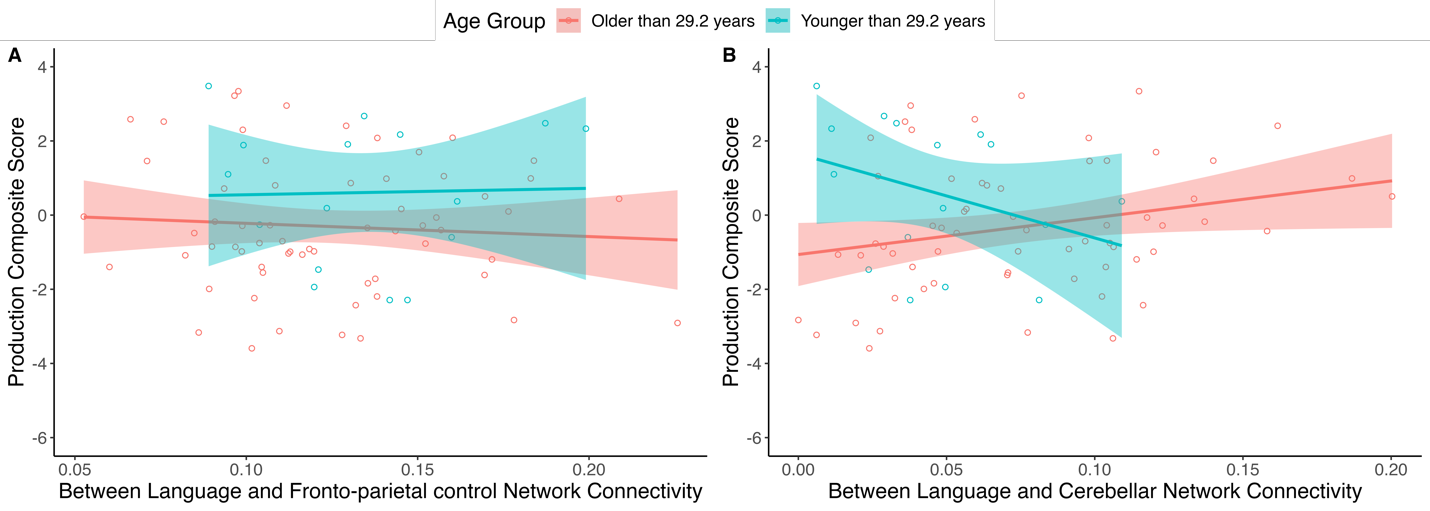


*Supplementary Figure 4* *Age-related differences in the relationship between language production composite scores and between network connectivity. A) shows a similarity across age groups in their relationship between the production score and between language and fronto-parietal control network connectivity. B) shows that in younger adults, weaker between language and cerebellar network connectivity was associated with higher production score.*
